# A Case Study on Addressing Complex Load Imbalance in OpenMP

**DOI:** 10.1007/978-3-030-58144-2_9

**Published:** 2020-08-03

**Authors:** Fabian Orland, Christian Terboven

**Affiliations:** 8 Texas Advanced Computing Center (TACC), Austin, TX USA; 9 Lawrence Livermore National Laboratory, Livermore, CA USA; 10 Texas Advanced Computing Center (TACC), Austin, TX USA; 11 RWTH Aachen University, Aachen, Germany; Chair for Computer Science 12 - High-Performance Computing, RWTH Aachen University, Aachen, Germany

**Keywords:** OpenMP, Load balance, Dynamic load balancing, Tasking, Nested parallelism, GMRES, Convergence, SPMD

## Abstract

Load balance is an important factor that fundamentally impacts the scalability of any parallel application. In this paper we present a case study to address a complex load imbalance related to the convergence behavior of the parallel SPMD implementation of a GMRES solver used in a real world application in the field of computational fluid dynamics. In order to tackle this load imbalance in OpenMP we illustrate different approaches involving the use of nested tasks as well as nested parallel regions. Furthermore, we evaluate these approaches on a small kernel program extracted from the original application code and show how the load balance is affected by each of these approaches.

## Introduction

Currently the largest HPC systems listed in the top500 list [3] offer hundreds of thousands or even millions of cores. In order to scale any scientific application code to such large scales the application has to efficiently utilize every available hardware resource. When doing strong scaling measurements of an application the fundamental assumption is that the code can be perfectly parallelized which in reality is not always the case [5].

For shared-memory systems the OpenMP [10, 14] programming interface offers a range of concepts for load balancing such as different loop schedules or the task construct. A static loop schedule divides the loop iterations in chunks of equal size and assigns these chunks to threads in a round-robin fashion. Using dynamic schedules each thread requests a chunk of iterations and upon completion requests another chunk until all loop iteration have been carried out. With a guided schedule the size of these chunks varies. First some large chunks are created and then for further chunks the size is decreased steadily. The OpenMP task construct allows user-defined chunks of work to be completed asynchronously which can already lead to a good load balance.

In this paper we want to raise attention to a special kind of load imbalance that can occur in the SPMD implementation of iterative solvers and is complex to tackle. We discovered a scenario in which loop scheduling cannot be applied and splitting the original problem into smaller subproblems executed as tasks increases the amount of computation to be performed instead of reducing it.

## Related Work

In order to quantify load imbalances different metrics have been established. The POP project [2], an EU Centre of Excellence in HPC, defines the load balance efficiency as the ratio between the average computation time across all execution units and the maximum computation time across these. For example, a load balance efficiency of 75% indicates that 25% of the available hardware resources are not properly utilized.

Unfortunately, this metric does not give any insight into the actual load distribution. Different distributions can have the same load balance efficiency but need to be tackled in different ways in order to improve the load balance. For example, it might make a difference if there are many slightly overloaded execution units or only a few but therefor heavily overloaded. Hence, Pearce et al. [15] propose to also take statistical moments like standard deviation, skewness and kurtosis into account. We use these metrics to quantify our load balance problem in this paper.

The OpenMP load balancing constructs have already been studied in the past. Durand et al. proposed an adaptive schedule which dynamically determines the chunk size depending on the utilization of the machine resources and also takes NUMA affinity information into account [13]. Recently, Ciorba et al. [8] investigated the state-of-the-art loop scheduling techniques. However, in our work dynamic loop scheduling cannot be applied because the application statically creates a single work load for each thread. We show that splitting these work loads into multiple smaller units, which could then be scheduled dynamically, will actually increase the overall runtime.

In the field of social and networking analysis Adcock et. al. used tasks to split up the computation of the $\delta$-hyperbolicity into multiple levels of small chunks which yielded good load balancing at a scale of 1000 threads [4]. Recently, tasks have been used successfully to balance the work in a Density Matrix Renormalization Group algorithm [9]. Identifying different kinds of tasks as well as assigning higher priorities to large tasks compared to small tasks lead to a more balanced execution. Based on the idea of using nested parallelism as discussed by Royuela et al. [16] we show how the load balance can be improved by implementing nested tasks as well as nested parallel regions into the code.

## Complex Load Imbalance

During our studies on the CalculiX [1, 12] application code we discovered an interesting and complex kind of load imbalance. Further investigation revealed that the issue is related to the GMRES solver [17]. Here the GMRES implementation provided by the SLATEC project is used [7, 18]. Hence, in this section we will first give a brief summary of the parallel GMRES implementation first and then present the structural pattern that we found in the code leading to a load imbalance.

### Generalized Minimal Residual Method

The generalized minimal residual method (GMRES) originally developed by Yousef Saad and Martin H. Schultz in 1986 [17] is a widely used iterative method to solve linear systems of the form $A \textit{\textbf{x}} = \textit{\textbf{b}}$, where *A* is a nonsymmetric matrix. The main idea is to create a Krylov subspace $\mathcal {K}(\textit{\textbf{v}}_1) = span \{ \textit{\textbf{v}}_1, A \textit{\textbf{v}}_1, \dots , A^{m} \textit{\textbf{v}}_1 \}$ using Arnoldi’s method [6] and approximate the exact solution of the linear system by a vector in that subspace which minimizes the residual norm. This process is repeated until the solution convergences up to a certain tolerance.

### Parallel GMRES

In the CalculiX code the governing equations of the Computational Fluid Dynamics problem are discretized using the finite volume method [11]. The simulation is discretized in time by individual timesteps called increments. To obtain a steady state solution for the primary variables, such as velocity, temperature and pressure, several inner iterations are performed in which the physical conservation laws are solved in their transient form until they converge to a steady solution [12]. In each of these inner iterations multiple nonsymmetric linear equation systems have to be solved. The size of these systems is determined by the number of elements the mesh is composed of. Typically, millions of elements are used to discretize a given geometry. In order to solve these large systems the GMRES method is applied in parallel as follows:

Consider a single of these systems at an inner iteration *k* given by

1 $$\begin{aligned} A[\textit{\textbf{u}}^{k-1}] \textit{\textbf{u}}^k = \textit{\textbf{b}}[\textit{\textbf{u}}^{k-1}], \end{aligned}$$

where $\textit{\textbf{u}}^k \in \mathbb {R}^n$ is the velocity field at the end of the inner iteration *k*. Both the left hand side matrix $A \in \mathbb {R}^{n \times n}$ and the right hand side vector $\textit{\textbf{b}} \in \mathbb {R}^n$ depend on the solution of the previous inner iteration $\textit{\textbf{u}}^{k-1}$. Let *T* be the number of threads used for the parallelization. The matrix *A* gets subdivided into a $T \times T$ grid of submatrices $A_{i,j} \in \mathbb {R}^{nblk \times nblk}$ with $i,j \in \{ 1,2, ..., T\}$ and the vectors $\textit{\textbf{u}}$ and $\textit{\textbf{b}}$ are split correspondingly into

2 $$\begin{aligned} A = \begin{pmatrix} A_{1,1} &{} \dots &{} A_{1,T} \\ \vdots &{} \ddots &{} \vdots \\ A_{T,1} &{} \dots &{} A_{T,T} \end{pmatrix}, ~~~ \textit{\textbf{u}} = \begin{pmatrix} \textit{\textbf{u}}_1 \\ \vdots \\ \textit{\textbf{u}}_T \end{pmatrix}, ~~~ \textit{\textbf{b}} = \begin{pmatrix} \textit{\textbf{b}}_1 \\ \vdots \\ \textit{\textbf{b}}_T \end{pmatrix}, \end{aligned}$$

where the size of each submatrix $A_{i,j}$ is determined as $nblk = \lceil \frac{n}{T} \rceil$. Splitting the system in this fashion leads to *T* smaller subsystemsHowever, these systems are not independent as they are still connected by the various $\textit{\textbf{u}}_i^k$ for $i \in \{1, 2, ..., T\}$. Thus, by assuming that the solution $\textit{\textbf{u}}^k$ only changes slightly between each iteration one can approximate $\textit{\textbf{u}}^k \approx \textit{\textbf{u}}^{k-1}$ and reorder the system to yieldAs a result there are now *T* independent, smaller subsystems of the form

3 $$\begin{aligned} \tilde{A}_t \varvec{\tilde{u}}_t = \tilde{b}_t, \end{aligned}$$

where we have $\tilde{A}_t = A_{t,t}[\textit{\textbf{u}}^{k-1}] \cdot \textit{\textbf{u}}^k_t$, $\varvec{\tilde{u}}_t = \textit{\textbf{u}}^k_t$ and $$\varvec{\tilde{b}} = \textit{\textbf{b}}_t - \sum _{\begin{array}{c} i = 1 \\ i \ne t \end{array}}^T A_{t,i}[\textit{\textbf{u}}^{k-1}] \cdot \textit{\textbf{u}}^{k-1}_i$$. So in order to solve the whole system in parallel each of these smaller subsystems is solved by a single thread using a serial GMRES implementation.

### Convergence Dependent Load Imbalance

When we studied the CalculiX application we noticed a pattern occurring over the course of the whole simulation, in which one thread takes significantly longer to finish its GMRES computation than the other threads. In order to analyse this issue in more detail we isolated the solution of one of these systems and extracted a small kernel program by saving input and output data like matrices and vectors to file. In the original CalculiX code worker threads are forked and joined using the pthread API. We translated this equally into using an OpenMP parallel region so that we can use OpenMP constructs like tasks to implement solutions tackling the load imbalance later on.

**Fig. 1.**
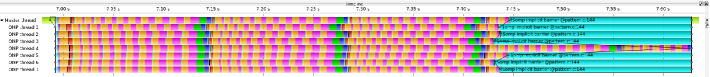
Trace visualization of our GMRES kernel program using 8 OpenMP threads. Different colors correspond to different operations performed by the GMRES solver (Color figure online).

**Table 1.** Load balance metrics obtained with our reference kernel using 8 to 48 OpenMP threads. We measured POP load balance efficiency, standard deviation, skewness and kurtosis based on runtime and GMRES iterations.

	threads	POP eff	std. dev	skewness	kurtosis
runtime	8	73%	0.065	2.192	2.959
	16	71%	0.024	3.555	10.790
	32	67%	0.012	3.531	14.152
	48	60%	0.013	4.144	17.011
iterations	8	72%	4.841	2.227	3.039
	16	69%	3.849	3.493	10.492
	32	67%	3.211	3.803	14.647
	48	68%	3.041	4.010	15.803

Figure 1 shows a trace of our kernel program executed with 8 OpenMP threads. We will refer to the master thread as thread 0 to match the numbering of threads in the trace correctly. On first sight the load imbalance becomes directly apparent because thread 4 takes significantly longer to finish compared to the others. We color coded different important subroutines of the GMRES implementation in the trace to highlight the iterative structure of this solver. One iteration consists mostly of applying a preconditioner in msolve (yellow) followed by a matrix vector product matvec (orange) and the orthogonalization dorth (pink) of the resulting vector. After 10 of such sequences the residual is calculated in drlcal (green) and the method is restarted in case the residual is not low enough. Based on this information we can count the number of GMRES iterations that each thread performs in the trace. While most of the threads obtain a converged solution after 31 or 32 iterations thread 4 requires 46 iterations. In order to quantify the load imbalance in our kernel program we measured load balance efficiency, standard deviation, skewness and kurtosis. All of these metrics are computed based on the runtime of the threads as well as on the number of GMRES iterations they perform. The results are shown in Table 1. First of all, we can verify that the kernel indeed has a significant load imbalance. The load balance efficiency based on runtimes ranges from 60% with 48 threads to 73% with 8 threads. Comparing these values to the load balance efficiency obtained based on GMRES iterations reveals a correlation between them. In most cases the values are nearly the same, except for the execution with 48 threads. Here we now have two slow threads while for the other executions we only have one. Furthermore, in both slow threads we find a single call to the subroutine dorth which suddenly takes much longer to complete compared to all other calls to this routine in the whole execution. This leads to lower efficiency value based on runtime than on GMRES iterations.

The standard deviation is not really comparable because runtime and iterations are measured in different units and have different magnitudes. However, skewness and kurtosis can be compared. We recognize that we get nearly the same results for all numbers of threads when comparing values based on runtimes and iterations. A positive skewness indicates that only a few number of threads are overloaded. The high kurtosis values indicate that variances are caused by infrequent extreme changes, i.e. by the one (or two) slow thread(s).

Even though the subsystems that each thread has to solve are of equal size in our case, the different convergence behavior leads to a load imbalance. This kind of imbalance is hard to tackle because it is difficult to predict the required number of GMRES iterations prior to the execution.

## Load Balance Strategies

In order to improve the load balance of our parallel GMRES kernel program we implemented different ideas. The first idea uses OpenMP tasks to create multiple smaller subsystems to be solved in parallel. The second idea creates tasks conditionally only in the unbalanced phase of the execution. Lastly, the third idea is similar to the second one but instead of conditionally creating tasks it uses nested parallel regions in the unbalanced phase of the execution. In the following subsections each idea will be presented in more detail.

### Tasking

In the first approach we use the OpenMP task construct. The idea is relatively simple: Instead of creating only a single subsystem to be solved by each thread we create multiple. Each subsystem is expressed as one OpenMP task. Depending on how many tasks we create a single task will shrink meaning that the subsystem to be solved will be smaller. In case a thread encounters a subsystem that converges slower than the other ones, the other threads can be kept busy by the OpenMP runtime scheduling another task from the pool of tasks to them. As a result we expect the total execution to be more balanced among the threads than with the original work distribution.

### Conditional Nested Tasks

Our second approach directly tackles the unbalanced part of the execution. In our example (Fig. 1) this is the point at which all threads except thread 4 are finished with their computation. The idea is to conditionally split the remaining work in the slow thread into tasks that can be executed by the idling threads as well.

Therefore, we identified some subroutines of the GMRES solver that can be potentially parallelized. In all of these subroutines most work is done in some loops that can trivially be parallelized. We illustrate the implementation of our approach by the example of the matvec subroutine.

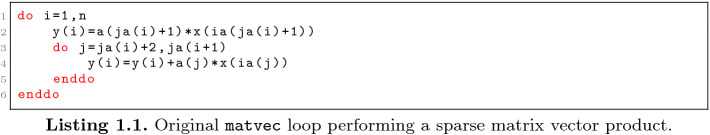



The original Fortran code is shown in Listing 1.1. It shows the main loop that iterates over the rows of a sparse matrix a stored in CSR format. For each row of a it computes the inner product of that row with the column vector x and stores the result in the corresponding location in the result vector y. The computation for each row is completely independent from the other rows so we can easily parallelize the outer do loop.

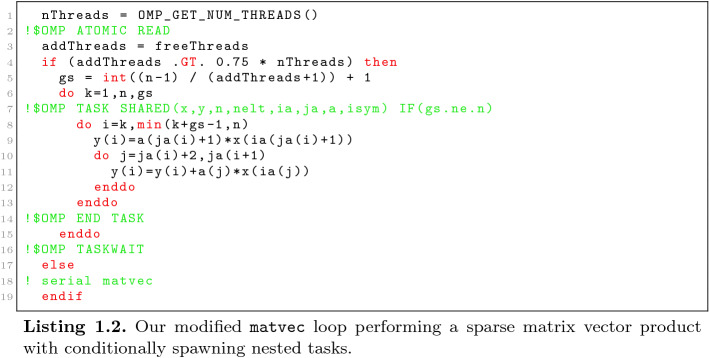



Our modifications to the code to implement our load balancing strategy are shown in Listing 1.2. We keep track of the number of threads that already finished their GMRES computation by atomically increasing a global variable freeThreads. Before we start computing the matrix vector product we atomically read this variable and save it in the variable addThreads (line 3).

If more than 75% of all threads are already finished with their own GMRES computation we consider to be in the unbalanced phase of the execution (line 4). In our example with 8 threads this corresponds to at least 7 threads that are already finished. Based on the number of freeThreads we determine the grainsize (gs) (line 5) in order to split the outer do loop into as many OpenMP tasks as there are freeThreads, including the slow running thread of course (lines 6–15). So each task corresponds to a number of rows the matrix vector product is performed on.

If less than 75% of all threads have finished their own computation we consider to still be in the balanced phase. In this case we default to the original serial matvec implementation (line 18). This should avoid any overhead the task creation may introduce when we know that only one task would be created anyways.

### Conditional Nested Parallel Region

Our third and last approach is very similar to our second approach. It also conditionally splits the remaining work of the slow thread in the unbalanced phase between the other threads. However, as there is a certain overhead of creating nested tasks we use nested parallel regions as an alternative in this approach. Again we present the implementation of our approach by the example of the matvec subroutine. For the original Fortran code we refer to Listing 1.1.

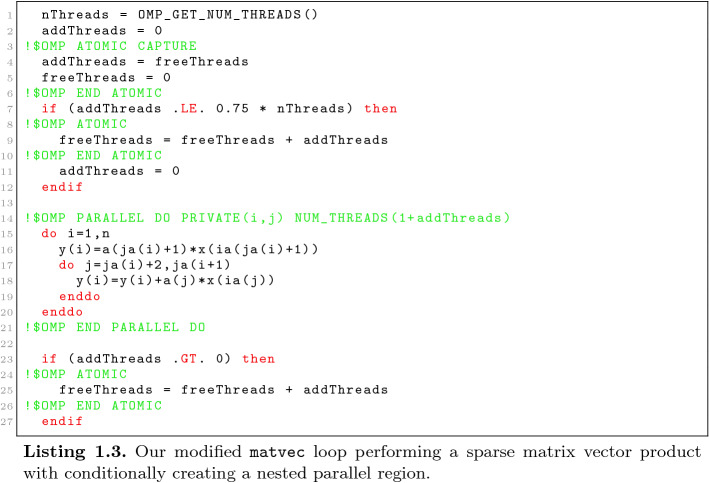



Our modifications in order to implement this third approach are shown in Listing 1.3. Again we track the number of threads that already completed their own GMRES computation in a global variable freeThreads. Before performing the matrix vector product loop we atomically read the value of freeThreads and save it in another variable addThreads indicating how many additional threads we can use for the following computation (line 4). In this approach we have to make sure that we also set freeThreads to zero to make sure no other slow thread sees the freeThreads and would create additional nested threads as well (line 5). Then again we evaluate our condition to determine if we are already in the unbalanced phase or not (line 7).

If we are in the balanced phase then we logically release all additional threads that we would have used to speed up computation on the current thread by performing an atomic update on freeThreads (line 9) and setting addThreads to zero. Conversely, if we are in the unbalanced phase addThreads holds the value of other idling threads at this point in the computation (line 13).

It follows the main do loop that performs the matrix vector product. This time we embedded it into a nested parallel region (lines 14–21). Depending on the evaluation of our condition to distinguish between the balanced and unbalanced phase this region will be executed with a different number of threads (line 14). If we are in the balanced phase $\texttt {addThreads} = 0$ and the parallel region will only be executed by the current thread. However, if we are in the unbalanced phase $\texttt {addThreads} > 0$ and the parallel region will be executed by the current thread together with some additional threads depending on how many are currently idling. In our example with 8 threads this will be all the other 7 threads.

After the parallel region has been executed we need to make sure to logically release the additional threads again by performing an atomic update on freeThreads (line 25). Of course this only needs to be done if we have really used them so only if $\texttt {addThreads} > 0$ (line 23). Otherwise we can save the atomic update operation.

## Results

In this section we will present some performance results obtained with our kernel program. For each of our three implemented approaches we will show how it affects the load balance of our kernel.

All measurements were done on one node of the CLAIX-2018 cluster system of RWTH Aachen University. Such a node is a two-socket system equipped with two Intel Xeon Platinum 8160 processors. Each processor provides 24 cores running with a clock frequency of 2.1 GHz and 192 GB of memory. In order to fully exploit the memory bandwidth of this NUMA architecture threads are placed onto cores according to the policy of KMP_AFFINITY=scatter. To compile the programs we used the Intel Fortran compiler version 19.0.1.144 2018 which also provides an OpenMP runtime. For performance analysis we used Score-P 6.0, Cube 4.5 (release preview) and Vampir 9.8.0.

### Tasking

Performance results obtained with the version of our kernel program that implements tasking as described in Sect. 4.1 are shown in Table 2. The kernel was executed with 8 OpenMP threads. We created a different number of tasks per thread and measured the load balance, the number of instructions executed as well as the time spent inside the GMRES kernel accumulated over all threads. First of all we recognize that the more tasks we create the closer the load balance approaches 100%. So in terms of load balancing this approach is nearly optimal. However, this approach also comes with a huge drawback. Already if we create 5 tasks per thread the accumulated time spent inside the kernel over all threads increases by 62%. Though with 10 tasks per thread this runtime only increases by 51%. In the extreme case of creating 150 tasks per thread the time spent in the kernel is 87% higher than in the reference case of using just 1 task per thread. In all cases where the runtime increases there are also more instructions executed than in the reference case. This clearly indicates that the convergence of the GMRES method plays an important role. The systems to be solved are much smaller than in the reference case. For example, when creating 150 tasks per thread an individual system is only of size $1280 \times 1280$ compared to $192000 \times 192000$ in the reference case. Unfortunately, overall more work has to be done to obtain the same solution. So this approach does not improve the load balance in a sensible way.

**Table 2.** Performance results of our tasking approach with 8 OpenMP threads.

Tasks per thread	Load balance	instructions	time (accumulated)
1	74%	${3.83}\times {10^{10}}$	3.73 s
5	89%	${5.63}\times {10^{10}}$	6.05 s
10	96%	${5.71}\times {10^{10}}$	5.63 s
50	99%	${6.61}\times {10^{10}}$	6.59 s
100	99%	${6.41}\times {10^{10}}$	6.76 s
150	99%	${6.25}\times {10^{10}}$	6.98 s

**Fig. 2.**
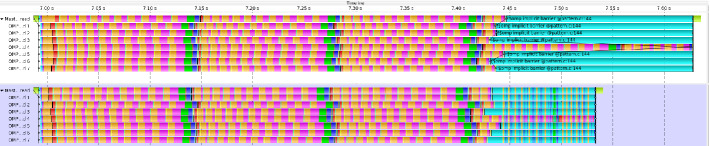
Trace comparison of our reference GMRES kernel (top) and the conditional nested tasks version (bottom) both using 8 OpenMP threads.

### Conditional Nested Tasks

In order to evaluate our second approach to tackle the load imbalance in our kernel we obtained a trace of the execution. A comparison between the original kernel and the one implementing conditional nested tasks is shown in Fig. 2. The balanced phase of the execution is almost identical in both traces. However, the unbalanced phase is significantly shorter using nested tasks compared to the reference. While thread 4 originally finished after roughly 630 ms it is now already finished after roughly 540 ms. This is a speedup of 1.16 compared to the reference. Moreover, the load balance has improved to 89%(+16%). The standard deviation in the runtimes is almost halved. This indicates that indeed the runtime on the slow thread got shorter while the runtimes on the other threads get longer because they now additionally spend time with computations inside the nested tasks. So overall the runtimes are now closer together than before. The skewness and kurtosis are also slightly lower than in the reference execution. This means the characteristics of the runtime distribution among the threads are still the same. We still have only one slow thread. However, this thread is now faster.

We obtained similar results for executions with a higher number of threads. Table 3 shows load balance metrics obtained for thread numbers ranging from 8 to 48. By comparing these results with the reference results shown in Table 1 we can see that the load balance efficiency is improved in all cases. While for 8 and 16 threads we yield an improvement of 16% we only get 11% with 32 threads and 4% with 48 threads. The same trend can be observed for the speedup factors. With 8 and 16 threads we yield a speedup of 1.16 and 1.18 respectively. However, with 32 threads only a speedup of 1.09 is obtained. Even worse when running with 48 threads the runtime is still the same as in the reference execution. This might be an impact of the overhead when frequently spawning nested tasks because each individual task is quite small and only operates on vectors with roughly 667 elements. The statistical moments are all slightly lower compared to the reference. Again this means that in all cases the runtime characteristics of the load imbalance stay the same. But the individual runtimes of each thread are now closer to the average.

**Table 3.** Load balance metrics obtained with our kernel implementing nested tasks using 8 to 48 OpenMP threads. We measured POP load balance efficiency, standard deviation, skewness, kurtosis and the speedup compared to the reference kernel.

	threads	POP eff	std. dev	skewness	kurtosis	speedup
nested tasks	8	89%	0.035	2.029	2.557	1.16
	16	87%	0.012	3.349	9.808	1.18
	32	78%	0.009	3.033	11.691	1.09
	48	64%	0.013	4.119	16.670	1.00

Finally, we also verified our results. In all cases the converged solution is equal to the original solution in the reference case with respect to machine precision (16 digits). The number of GMRES iterations that each thread performs have also not changed.

**Fig. 3.**
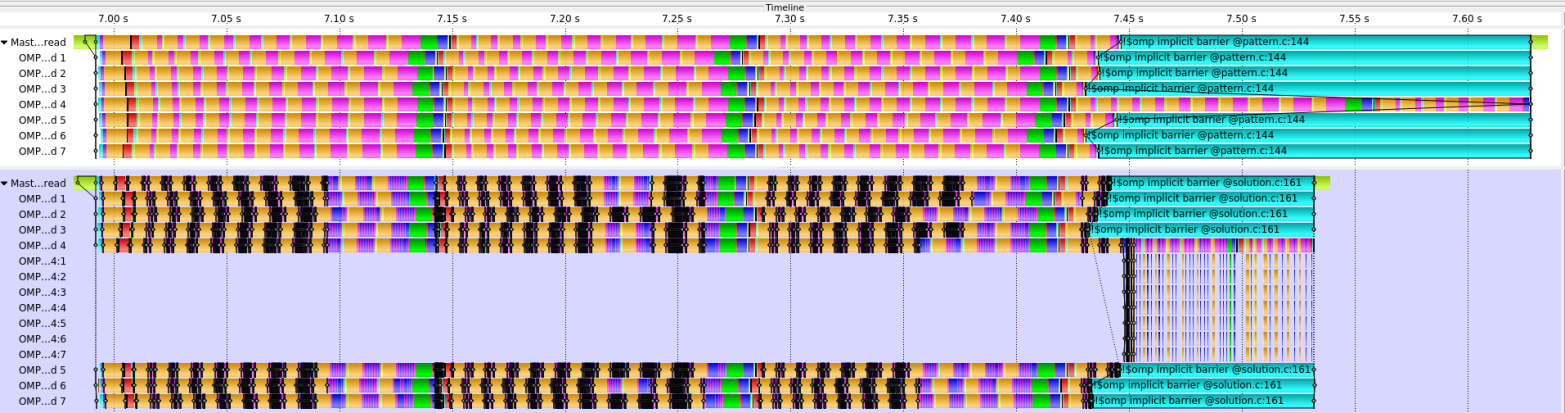
Trace comparison of our reference GMRES kernel (top) and the conditional nested parallel regions version (bottom) both using 8 OpenMP threads.

### Conditional Nested Parallel Region

A comparison between the original kernel and the one implementing conditional nested parallel regions is shown in Fig. 3. In the balanced phase we do not recognize any differences between the reference execution and the execution with nested parallel regions, except that these regions are visible in the trace even when they are executed by just one thread. However, in the unbalanced phase the runtime of the kernel is significantly shorter. Thread 4 obtains the converged solution after roughly 540 ms. This is a speedup of roughly 1.16 compared to the reference execution. The load balance is also significantly improved and is now at 88% (+15%). Furthermore, the statistical moments are also improved. The standard deviation almost got halved. This means that the variance in the individual runtimes of the threads are now smaller. The skewness is still positive and only slightly lower than before which shows that there is still only one overloaded thread. The kurtosis is also only slightly lower indicating that there are still infrequent large variances in the runtimes caused by the one slow thread.

Similar results are obtained with higher numbers of threads, ranging from 8 to 48, as shown in Table 4. In all cases the load balance efficiency is improved. Using 8 and 16 threads we yield an improvement of 15% and 16% respectively. However, with 32 threads we only yield a plus of 8%, which is also 3% less than with nested tasks. Using 48 threads we only get 3% improvement. The speedup values show a similar behavior. With 8 threads we get a speedup of roughly 1.16 which is identical to the nested task approach. But with 16 or more threads the nested parallel regions approach becomes a little bit slower than nested tasks. Using 16 threads we yield a speedup of 1.13 which is 5% slower than nested tasks. With 32 threads the speedup is only 1.02 and 7% slower than nested tasks. This becomes worse when using 48 threads. Here we yield a speedup of 0.95 which is a 5% slowdown compared to the reference execution. The statistical moments are all slightly lower compared to the reference case. However, they have still the same order of magnitude and are all positive. The similar skewness and kurtosis imply that still the load imbalance is caused by one overloaded thread.

**Table 4.** Load balance metrics obtained with our kernel implementing nested regions using 8 to 48 OpenMP threads. We measured POP load balance efficiency, standard deviation, skewness, kurtosis and the speedup compared to the reference kernel.

	threads	POP eff	std. dev	skewness	kurtosis	speedup
nested regions	8	88%	0.034	2.165	2.883	1.16
	16	87%	0.015	3.461	10.087	1.13
	32	75%	0.011	3.218	11.350	1.02
	48	63%	0.015	3.734	13.424	0.95

Moreover, the kernel still computes the correct solution. The converged solution is identical with the original one up to machine precision (16 digits). The number of GMRES iterations performed by each thread also remains the same.

In order to execute the kernel with nested parallel regions correctly the environment needs to be configured in a special way. First of all, we set OMP_NESTED = 1 to enable nested parallelism. Furthermore, we set KMP_HOT_TEAMS_MODE = 1 which will keep the nested threads in the team for faster reuse as multiple nested regions are quickly executed one after another. Related to this we also set KMP_BLOCKTIME = 0 which causes threads to instantly go to sleep state instead of waiting the default 200 ms after completing the execution of a parallel region. On the one hand this makes sure that outer level threads do not spend cpu time with idling. On the other hand this global environment variable also affects the nested threads, so that they will also instantly go to sleep state after executing a nested parallel region. Since we are rapidly executing lots of nested regions it would be much better if the blocktime could be set for each nesting level separately. Unfortunately, this is not possible with the current Intel OpenMP runtime.

Lastly, we pinned the kernel to a set of physical cores corresponding to the KMP_AFFINITY=scatter setting using taskset. The number of cores is equal to the number of outer level threads the kernel is executed with. Otherwise the nested threads could be scheduled on one of the remaining free physical cores of our system if there are some, for example when running with only 8 threads. However, the intent of our implementation is to mimic a similar behavior as with nested tasks. So by restricting the execution to as many physical cores as there are threads initially, we make sure that nested threads can only be scheduled to the same set of physical cores as the outer level threads.

## Future Work

Our current approach using nested parallelism has some drawbacks. Currently, the condition when to trigger the nested parallelism is hard-coded into the subroutines of the GMRES solver. Nested tasks or threads are spawned as soon as more than 75% are idling. For the presented load imbalance, where mostly only a single thread is heavily overloaded, this condition works quite well. However, for other cases with multiple slow threads it might not be suitable. Hence, we would like to also investigate arbitrary thresholds to trigger nested parallelism.

Moreover, the results presented in this paper focus only on the execution of a small kernel program extracted from the CalculiX application. Our results on the kernel look promising to also speedup the whole CalculiX application as the presented load imbalance pattern can be found over the course of the whole simulation. Hence, we want to verify the applicability of our approach using the whole CalculiX application in the future.

After that it might be interesting to identify similar load imbalances in implementations of iterative methods other than GMRES. If the load imbalance is similar to the pattern presented in this paper we expect our approach to be applicable as well.

Finally, our approach tackles the load imbalance when it already occurred. Thus, we are also interested in investigating the root cause of this imbalance. If we know what the imbalance is caused by we could tackle it directly and avoid the need to spawn nested tasks or nested regions at all.

## Conclusions

In this work we presented a very special kind of load imbalance that can occur in the parallel implementation of iterative methods used to solve systems of linear equations in an SPMD fashion. If each thread solves an independent subsystem different convergence behavior of these systems may induce a load imbalance between the threads.

We identified such a pattern in the CalculiX code in which one thread consistently has to perform more solver iterations than all the other threads thus reducing the load balance to 73% and implemented three different approaches to tackle the load imbalance: Splitting the problem into more even smaller subproblems leads to a perfectly balanced workload but also to a higher computational complexity and thus a longer runtime. Conditionally spawning nested tasks or nested parallel regions in the unbalanced phase of the execution both yield comparable results when running with 8 or 16 threads. Here we got a speedups between 1.13 and 1.18. But when running with 32 or 48 threads the kernel does not scale as well anymore so that in the worst case our approach slightly slowed the kernel down. Moreover, we investigated different statistical moments which indicate that the characteristics of the load imbalance are the same for all number of threads. In all cases we mostly have a single overloaded thread. Since the nested regions approach requires a special environment configuration, interferes with the thread scheduling of the OpenMP runtime and yielded a small application slowdown, we recommend to prefer nested tasks whenever possible.

Finally, our approach is directly implemented into the GMRES solver. It is independent from the CalculiX application code and can in general be used by other applications, that use the GMRES method in a similar way, as well.
